# Supplementary material for: Effect of Associated Bacteria GD1 on the Low-Temperature Adaptability of Bursaphelenchus xylophilus Based on RNA-Seq and RNAi
Source: Microorganisms. 2023 Feb 8;11(2):430. doi: 10.3390/microorganisms11020430 (PMC9961929; doi:10.3390/microorganisms11020430)
Supplement: Supplementary file 1 [file microorganisms-11-00430-s001.zip › Supplemental Figures.pdf]

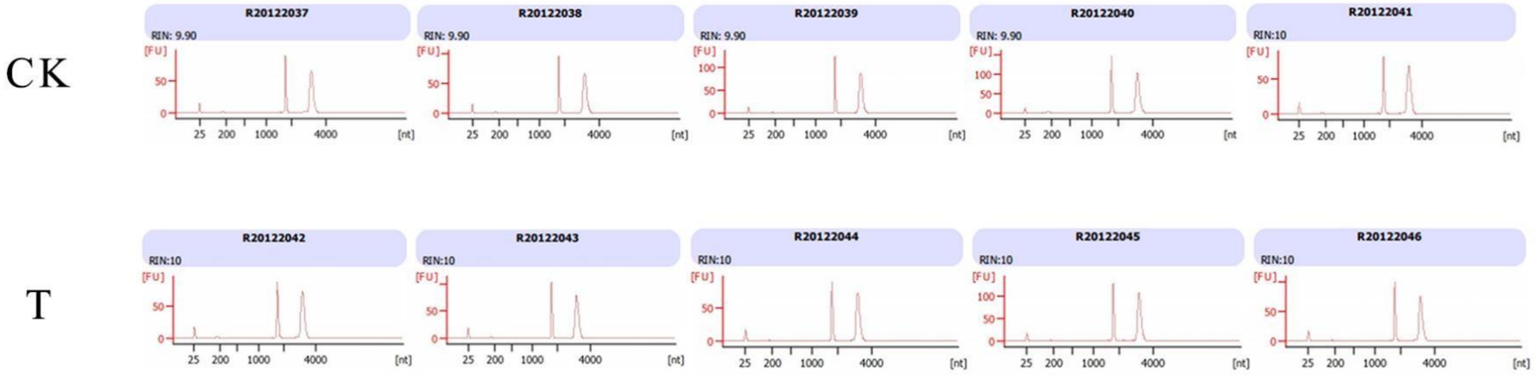

Figure S1 RIN of RNA. The two peaks represent the integrity of 18S and 28S ribosomal RNA

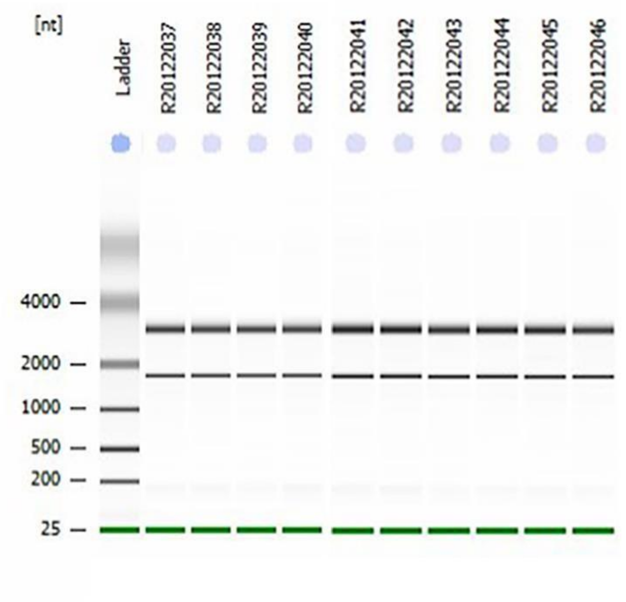

Figure S2 RNA agarose gel electrophoresis of AH23. The two bands represent the 18 s and 28 s ribosomal RNA

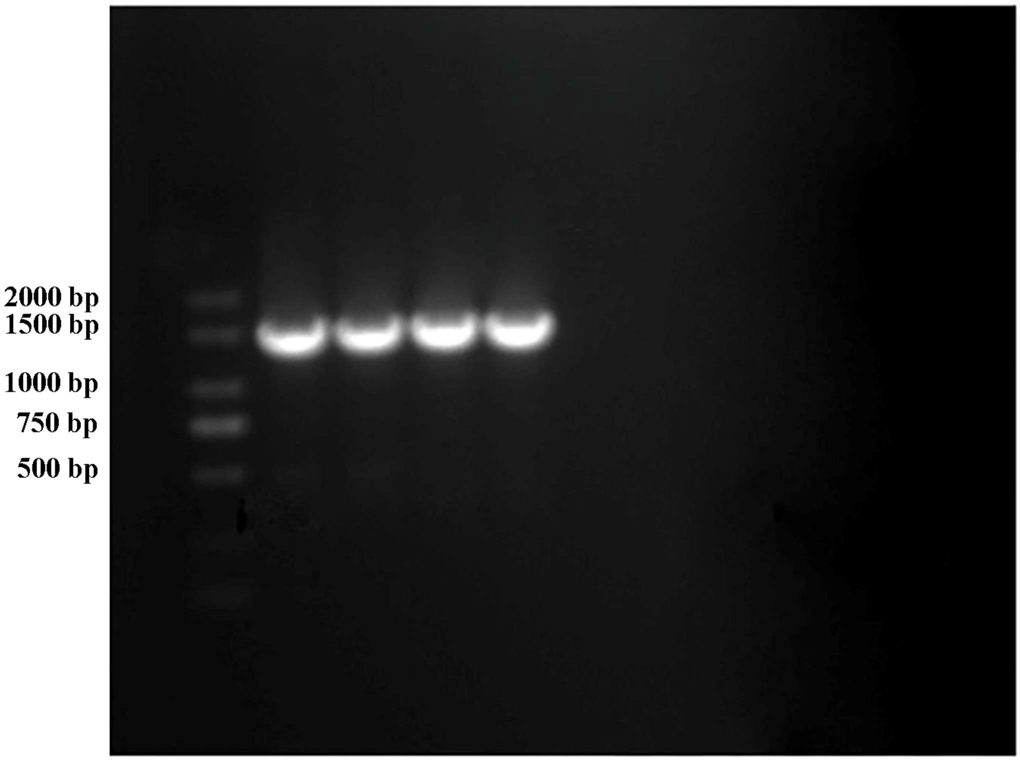

Figure S3 Agarose gel electrophoresis of *daf-11*
