# Supplementary material for: Effect of Associated Bacteria GD1 on the Low-Temperature Adaptability of Bursaphelenchus xylophilus Based on RNA-Seq and RNAi
Source: Microorganisms. 2023 Feb 8;11(2):430. doi: 10.3390/microorganisms11020430 (PMC9961929; doi:10.3390/microorganisms11020430)
Supplement: Supplementary file 1 [file microorganisms-11-00430-s001.zip › Supplemental Tables.pdf]

Supplemental Table S1. Transcriptome data filtering statistics

Sample	Raw Reads	Clean Reads (%)	Low Quality (%)
CK1	50525822	50396262 (99.74%)	118652 (0.23%)
CK2	53527442	53372122 (99.71%)	142554 (0.27%)
CK3	52847546	52700654 (99.72%)	133906 (0.25%)
CK4	51076532	50973434 (99.80%)	90740 (0.18%)
CK5	60860946	60737646 (99.80%)	107778 (0.18%)
T1	56105106	55977398 (99.77%)	115318 (0.21%)
T2	51497628	51393102 (99.80%)	92094 (0.18%)
T3	54178930	54066848 (99.79%)	100488 (0.19%)
T4	62043684	61924146 (99.81%)	105950 (0.17%)
T5	49375848	49269334 (99.78%)	94082 (0.19%)

Supplemental Table S2. Base mass analysis

Sample	Raw Date (bp)	Clean Date (bp)	GC (%)	Q30 (%)
CK1	7578873300	7558731199	47.97%	94.45%
CK2	8029116300	8005009127	47.87%	94.38%
CK3	7927131900	7904302467	47.88%	94.46%
CK4	7661479800	7645238033	47.83%	94.63%
CK5	9129141900	9109735921	47.89%	94.67%
T1	8415765900	8395816641	47.84%	94.64%
T2	7724644200	7708173716	47.89%	94.83%
T3	8126839500	8109219077	47.87%	94.44%
T4	9306552600	9287718767	47.59%	94.84%
T5	7406377200	7389614741	47.87%	94.53%

Supplemental Table S3. Ribosome alignment results

Sample	Clean Reads	Mapped Reads (%)	Unmapped Reads (%)
CK1	50396262	48218 (0.10%)	50348044 (99.90%)
CK2	53372122	43128 (0.08%)	53328994 (99.92%)
CK3	52700654	40810 (0.08%)	52659844 (99.92%)
CK4	50973434	36350 (0.07%)	50937084 (99.93%)
CK5	60737646	39892 (0.07%)	60697754 (99.93%)
T1	55977398	30198 (0.05%)	55947200 (99.95%)
T2	51393102	40406 (0.08%)	51352696 (99.92%)
T3	54066848	40926 (0.08%)	54025922 (99.92%)
T4	61924146	59926 (0.10%)	61864220 (99.90%)
T5	49269334	44794 (0.09%)	49224540 (99.91%)

Supplemental Table S4. Refer to the Genome Alignment Statistics

Sample	Clean Reads	Unique Mapped (%)	Multiple Mapped (%)
CK1	50396262	45791458 (90.95%)	520144 (1.03%)
CK2	53372122	48360903 (90.68%)	557124 (1.04%)
CK3	52700654	47889853 (90.94%)	533498 (1.01%)
CK4	50973434	46268443 (90.83%)	520664 (1.02%)
CK5	60737646	55415214 (91.30%)	627207 (1.03%)
T1	55977398	51019536 (91.19%)	580856 (1.04%)
T2	51393102	46863898 (91.26%)	540435 (1.05%)
T3	54066848	49205979 (91.08%)	550834 (1.02%)
T4	61924146	56440823 (91.23%)	633098 (1.02%)
T5	49269334	44917040 (91.25%)	500235 (1.02%)

Supplemental Table S5. Genetic testing statistics

Sample	Refer Genes (%)	Novel Genes (%)	Total Genes (%)
All	16082 (90.84%)	316 (100.00%)	16398 (91.00%)
CK1	14972 (84.57%)	289 (91.46%)	15261 (84.69%)
CK2	14996 (84.70%)	289 (91.46%)	15285 (84.82%)
CK3	15013 (84.80%)	290 (91.77%)	15303 (84.92%)
CK4	14969 (84.55%)	289 (91.46%)	15258 (84.67%)
CK5	15272 (86.26%)	294 (93.04%)	15566 (86.38%)
T1	15045 (84.98%)	292 (92.41%)	15337 (85.11%)
T2	15088 (85.22%)	294 (93.04%)	15382 (85.36%)
T3	14972 (84.57%)	291 (92.09%)	15263 (84.70%)
T4	15228 (86.01%)	300 (94.94%)	15528 (86.17%)
T5	15009 (84.78%)	284 (89.87%)	15293 (84.87%)

Supplemental Tables S6. KEGG pathway and pathway ID

NO.	Pathway	Pathway ID
1	Transcriptional misregulation in cancers	ko05202
2	EGFR tyrosine kinase inhibitor resistance	ko01521
3	MAPK signaling pathway	ko04010
4	Apoptosis - fly	ko04214
5	Mucin type O-glycan biosynthesis	ko00512
6	Sphingolipid signaling pathway	ko04071
7	Alanine, aspartate and glutamate metabolism	ko00250
8	Nitrogen metabolism	ko00910

9	Apoptosis	ko04210
10	Valine, leucine and isoleucine biosynthesis	ko00290
11	PI3K-Akt signaling pathway	ko04151
12	Protein digestion and absorption	ko04974
13	Renal cell carcinoma	ko05211
14	Neurotrophin signaling pathway	ko04722
15	D-Glutamine and D-glutamate metabolism	ko00471
16	Dorso-ventral axis formation	ko04320
17	Glycosphingolipid biosynthesis - lacto and neolacto series	ko00601
18	Riboflavin metabolism	ko00740
19	Arachidonic acid metabolism	ko00590
20	MicroRNAs in cancer	ko05206
21	Thiamine metabolism	ko00730
22	Tight junction	ko04530
23	Vasopressin-regulated water reabsorption	ko04962
24	HIF-1 signaling pathway	ko04066
25	Taurine and hypotaurine metabolism	ko00430
26	Glutathione metabolism	ko00480
27	mTOR signaling pathway	ko04150
28	Proximal tubule bicarbonate reclamation	ko04964
29	Biosynthesis of amino acids	ko01230
30	Long-term depression	ko04730
31	Lysosome	ko04142
32	Gap junction	ko04540
33	Insulin signaling pathway	ko04910
34	MAPK signaling pathway - fly	ko04013
35	Prostate cancer	ko05215
36	Huntington disease	ko05016
37	Rap1 signaling pathway	ko04015
38	Pancreatic secretion	ko04972
39	Focal adhesion	ko04510
40	Regulation of actin cytoskeleton	ko04810
41	Maturity onset diabetes of the young	ko04950
42	Longevity regulating pathway - mammal	ko04211
43	Pancreatic cancer	ko05212
44	Hippo signaling pathway - multiple species	ko04392
45	Circadian rhythm	ko04710
46	Chemical carcinogenesis	ko05204
47	Metabolic pathways	ko01100
48	Tyrosine metabolism	ko00350
49	Gastric cancer	ko05226
50	Cell cycle	ko04110
51	Hedgehog signaling pathway - fly	ko04341
52	Arginine biosynthesis	ko00220

53	One carbon pool by folate	ko00670
54	Spinocerebellar ataxia	ko05017
55	Progesterone-mediated oocyte maturation	ko04914
56	Cytosolic DNA-sensing pathway	ko04623
57	Thyroid cancer	ko05216
58	Central carbon metabolism in cancer	ko05230
59	mRNA surveillance pathway	ko03015
60	Drug metabolism - cytochrome P450	ko00982
61	Steroid biosynthesis	ko00100
62	Folate biosynthesis	ko00790
63	Vascular smooth muscle contraction	ko04270
64	Non-alcoholic fatty liver disease (NAFLD)	ko04932
65	Parathyroid hormone synthesis, secretion and action	ko04928
66	Purine metabolism	ko00230
67	Metabolism of xenobiotics by cytochrome P450	ko00980
68	Oocyte meiosis	ko04114
69	Fatty acid biosynthesis	ko00061
70	Antifolate resistance	ko01523
71	ECM-receptor interaction	ko04512
72	IL-17 signaling pathway	ko04657
73	Bladder cancer	ko05219
74	ABC transporters	ko02010
75	Renin-angiotensin system	ko04614
76	Axon regeneration	ko04361
77	Acute myeloid leukemia	ko05221
78	Long-term potentiation	ko04720
79	Estrogen signaling pathway	ko04915
80	Human papillomavirus infection	ko05165
81	Autophagy - other eukaryotes	ko04136
82	Autophagy - animal	ko04140
83	Melanoma	ko05218
84	Chemokine signaling pathway	ko04062
85	Herpes simplex infection	ko05168
86	Tuberculosis	ko05152
87	Carbohydrate digestion and absorption	ko04973
88	Glycine, serine and threonine metabolism	ko00260
89	RNA polymerase	ko03020
90	Hematopoietic cell lineage	ko04640
91	Natural killer cell mediated cytotoxicity	ko04650
92	Salivary secretion	ko04970
93	Mineral absorption	ko04978
94	Adrenergic signaling in cardiomyocytes	ko04261
95	Ascorbate and aldarate metabolism	ko00053
96	Axon guidance	ko04360

97	GnRH signaling pathway	ko04912
98	Ubiquinone and other terpenoid-quinone biosynthesis	ko00130
99	Other types of O-glycan biosynthesis	ko00514
100	Oxytocin signaling pathway	ko04921
101	Basal transcription factors	ko03022
102	VEGF signaling pathway	ko04370
103	cGMP - PKG signaling pathway	ko04022
104	Platelet activation	ko04611
105	Aldosterone-regulated sodium reabsorption	ko04960
106	Small cell lung cancer	ko05222
107	Bile secretion	ko04976
108	Cholesterol metabolism	ko04979
109	Alcoholism	ko05034
110	Pathways in cancer	ko05200
111	Alzheimer disease	ko05010
112	Lysine degradation	ko00310
113	GABAergic synapse	ko04727
114	TNF signaling pathway	ko04668
115	Systemic lupus erythematosus	ko05322
116	Amyotrophic lateral sclerosis (ALS)	ko05014
117	Shigellosis	ko05131
118	Ubiquitin mediated proteolysis	ko04120
119	Chronic myeloid leukemia	ko05220
120	Non-small cell lung cancer	ko05223
121	Hippo signaling pathway -fly	ko04391
122	Proteoglycans in cancer	ko05205
123	Leukocyte transendothelial migration	ko04670
124	Glycolysis / Gluconeogenesis	ko00010
125	Nucleotide excision repair	ko03420
126	TGF-beta signaling pathway	ko04350
127	Synaptic vesicle cycle	ko04721
128	cAMP signaling pathway	ko04024
129	Inositol phosphate metabolism	ko00562
130	Glyoxylate and dicarboxylate metabolism	ko00630
131	Propanoate metabolism	ko00640
132	Endometrial cancer	ko05213
133	Pentose and glucuronate interconversions	ko00040
134	Wnt signaling pathway	ko04310
135	Adherens junction	ko04520
136	Hippo signaling pathway	ko04390
137	Pathogenic Escherichia coli infection	ko05130
138	Sphingolipid metabolism	ko00600
139	Endocrine resistance	ko01522
140	Bacterial invasion of epithelial cells	ko05100

141	Ras signaling pathway	ko04014
142	Fluid shear stress and atherosclerosis	ko05418
143	Fatty acid elongation	ko00062
144	Aminoacyl-tRNA biosynthesis	ko00970
145	T cell receptor signaling pathway	ko04660
146	AGE-RAGE signaling pathway in diabetic complications	ko04933
147	Hepatitis C	ko05160
148	Glioma	ko05214
149	Viral carcinogenesis	ko05203
150	Endocrine and other factor-regulated calcium reabsorption	ko04961
151	Thyroid hormone synthesis	ko04918
152	Platinum drug resistance	ko01524
153	RNA transport	ko03013
154	Retinol metabolism	ko00830
155	Dopaminergic synapse	ko04728
156	Pyruvate metabolism	ko00620
157	Fc gamma R-mediated phagocytosis	ko04666
158	Hedgehog signaling pathway	ko04340
159	Gastric acid secretion	ko04971
160	Epithelial cell signaling in Helicobacter pylori infection	ko05120
161	AMPK signaling pathway	ko04152
162	Cardiac muscle contraction	ko04260
163	Glutamatergic synapse	ko04724
164	ErbB signaling pathway	ko04012
165	Renin secretion	ko04924
166	Breast cancer	ko05224
167	Yersinia infection	ko05135
168	Colorectal cancer	ko05210
169	Signaling pathways regulating pluripotency of stem cells	ko04550
170	Insulin secretion	ko04911
171	Porphyrin and chlorophyll metabolism	ko00860
172	Carbon metabolism	ko01200
173	Phosphatidylinositol signaling system	ko04070
174	Aldosterone synthesis and secretion	ko04925
175	Spliceosome	ko03040
176	Serotonergic synapse	ko04726
177	Hepatitis B	ko05161
178	Salmonella infection	ko05132
179	Circadian entrainment	ko04713
180	Amphetamine addiction	ko05031
181	Melanogenesis	ko04916
182	Glucagon signaling pathway	ko04922
183	Steroid hormone biosynthesis	ko00140
184	FoxO signaling pathway	ko04068

185	Protein processing in endoplasmic reticulum	ko04141
186	Neuroactive ligand-receptor interaction	ko04080
187	Cushing syndrome	ko04934
188	Inflammatory mediator regulation of TRP channels	ko04750
189	Endocytosis	ko04144
190	Glycerophospholipid metabolism	ko00564
191	Thermogenesis	ko04714
192	Glycerolipid metabolism	ko00561
193	Choline metabolism in cancer	ko05231
194	Thyroid hormone signaling pathway	ko04919
195	Fatty acid metabolism	ko01212
196	Insulin resistance	ko04931
197	Phagosome	ko04145
198	Oxidative phosphorylation	ko00190
199	HTLV-I infection	ko05166
200	Cellular senescence	ko04218
201	Retrograde endocannabinoid signaling	ko04723
202	Hepatocellular carcinoma	ko05225
203	Parkinson disease	ko05012
204	Human immunodeficiency virus 1 infection	ko05170
205	Peroxisome	ko04146
206	Drug metabolism - other enzymes	ko00983
207	Ribosome	ko03010

Supplemental Table S7. Sequences of the primers used in this study

Primer	Sequence（5’ → 3’）
q_0171500_F	CTTGTGGTCTTTGCAACCCG
q_0171500_R	TTTGCAAACTGCAGCGATGG
q_1454800_F	CCAGTCACCGAAGAGCCAAT
q_1454800_R	CGGTCACTTCCTCAGGGTTG
q_1389600_F	TTGTCGCCTCGGATTGTGAA
q_1389600_R	GCGACACAGATCCCACATGA
q_0345700_F	GGGTTTCTACCTTCGGATTGT
q_0345700_R	GGTCTTCCCACATAGGCTTAAT
q_0802300_F	ACGCGACAGCTCAAGAAA
q_0802300_R	TCGAACTCAACAAACGCAAAG
q_1604100_F	GCGGAATGCGAGAAAGAATTTA
q_1604100_R	CCGTGTGCCAAATGTTCATAG
q_0370100_F	GCAGAAATGGTGCAGCTAATG
q_0370100_R	TGTTCATGCTCTCCTCACAAA
q_1348900_F	GACGTGAACACCTTCGATGA
q_1348900_R	TGCCCATTTGTGCTCCATA
q_0845700_F	CAGGAAGTGCTGGGAAAGTAT
q_0845700_R	GTCGATGGAGTTGGCTATGATTA
q_0662700_F	GGTGTTGAAGGAGAAGGAAGAG
q_0662700_R	GGAATCAGATGAACCGGAGATT
q_*daf-11*_F	GACTTACTGGCTTGATGGGATAG
q_*daf-11*_R	CCTCGCGTCTTCTGGTATTG
Actin_F	GCAACACGGAGTTCGTTGTA
Actin_R	GTATCGTCACCAACTGGGAT
*daf-11*_F	CCAAAGGAAGAATACGCC
*daf-11*_R	ATTTTATTGCCCTTCTCG
M13F	GTAAAACGACGGCCAGT
M13R	CAGGAAACAGCTATGAC
ds_*daf-11*_1	GATCACTAATACGACTCACTATAGGGGCAGCACAACAATCGTTTATT
ds_*daf-11*_2	AATAAACGATTGTTGTGCTGCCCCTATAGTGAGTCGTATTAGTGATC
ds_*daf-11*_3	AAGCAGCACAACAATCGTTTACCCTATAGTGAGTCGTATTAGTGATC
ds_*daf-11*_4	GATCACTAATACGACTCACTATAGGGTAAACGATTGTTGTGCTGCTT
ds_gfp_1	GATCACTAATACGACTCACTATAGGGGGATGTCTCACATCTTGTTT
ds_gfp_2	AAACAAGATGTGAGACATCCCCCCTATAGTGAGTCGTATTAGTGATC
ds_gfp_3	AAGGGATGTCTCACATCTTGTCCCTATAGTGAGTCGTATTAGTGATC
ds_gfp_4	GATCACTAATACGACTCACTATAGGGACAAGATGTGAGACATCCCTT
